# Supplementary material for: Spatio-temporal dynamics of hand, foot and mouth disease in Malaysia, 2009–2019
Source: PLoS Negl Trop Dis. 2025 Jun 9;19(6):e0013174. doi: 10.1371/journal.pntd.0013174 (PMC12180618; doi:10.1371/journal.pntd.0013174)
Supplement: S1 Fig — The monthly proportion of EVA-71 positive samples among all EVA-71 or CVA16 positive samples (black points) with 95% exact binomial confidence intervals. A spline (shown in red) was fitted through the points using 28 knots (total data points/3). (PDF) [file pntd.0013174.s001.pdf]

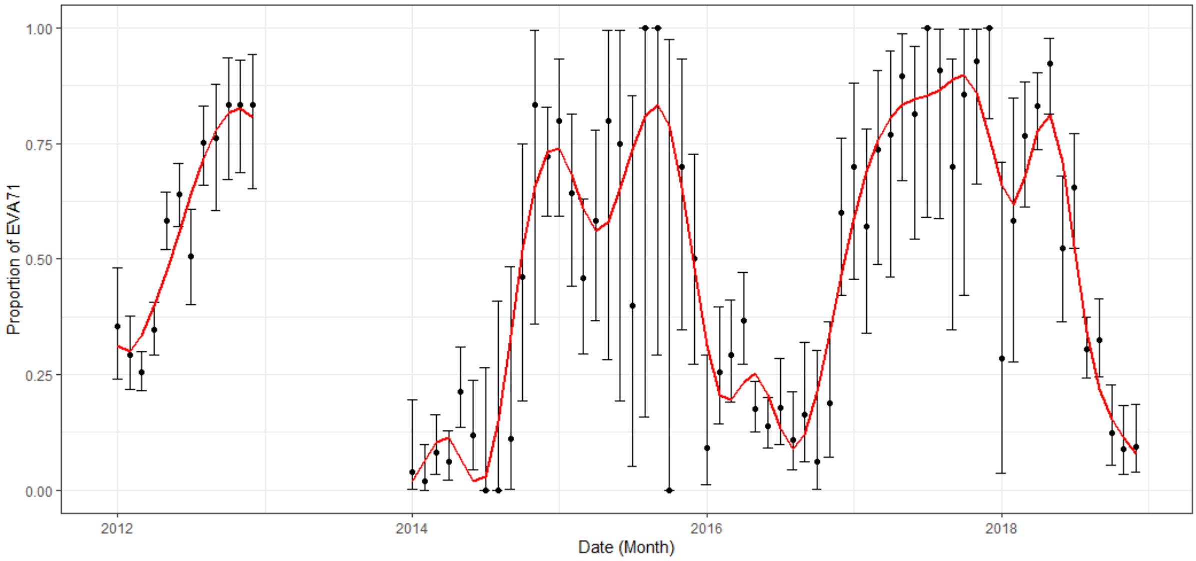

Figure S1. Proportion of EV-A71. The monthly proportion of EV-A71 positive samples among all EV-A71 or CVA16 positive samples (black points) with 95% exact binomial confidence intervals. A spline (shown in red) was fitted through the points using 28 knots (total data points / 3).
