# Supplementary material for: Spatio-temporal dynamics of hand, foot and mouth disease in Malaysia, 2009–2019
Source: PLoS Negl Trop Dis. 2025 Jun 9;19(6):e0013174. doi: 10.1371/journal.pntd.0013174 (PMC12180618; doi:10.1371/journal.pntd.0013174)
Supplement: S2 Fig — Daily number of hand-foot-and-mouth disease cases reported in the Federal Territory of Kuala Lumpur (top) and Sarawak (bottom) coloured by the school holiday periods in red. (PDF) [file pntd.0013174.s002.pdf]

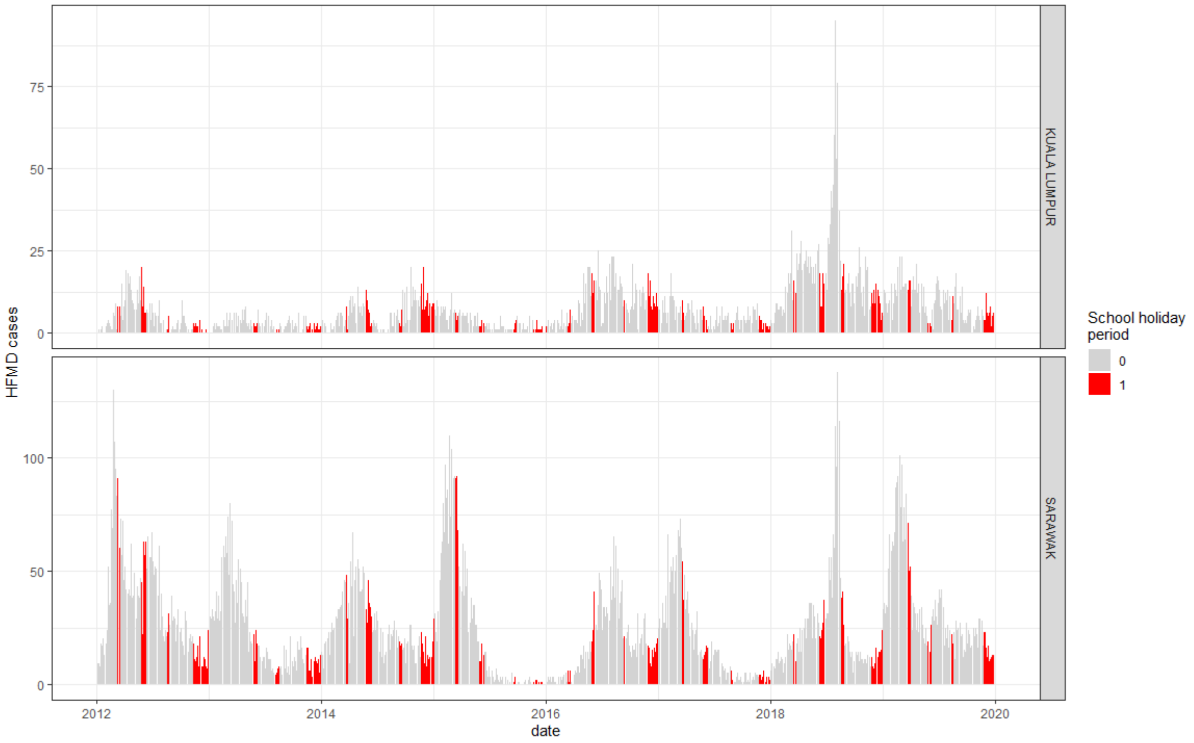

Figure S2. School holidays. Daily number of hand-foot-and-mouth disease cases reported in the Federal Territory of Kuala Lumpur (top) and Sarawak (bottom) coloured by the school holiday periods in red.
