# Supplementary material for: Spatio-temporal dynamics of hand, foot and mouth disease in Malaysia, 2009–2019
Source: PLoS Negl Trop Dis. 2025 Jun 9;19(6):e0013174. doi: 10.1371/journal.pntd.0013174 (PMC12180618; doi:10.1371/journal.pntd.0013174)
Supplement: S3 Fig — Meteorological variables for each day in 2012–2019 coloured for each state in Malaysia, arranged with states in Peninsular Malaysia in the left-hand panels and states in East Malaysia in the right-hand panels. (PDF) [file pntd.0013174.s003.pdf]

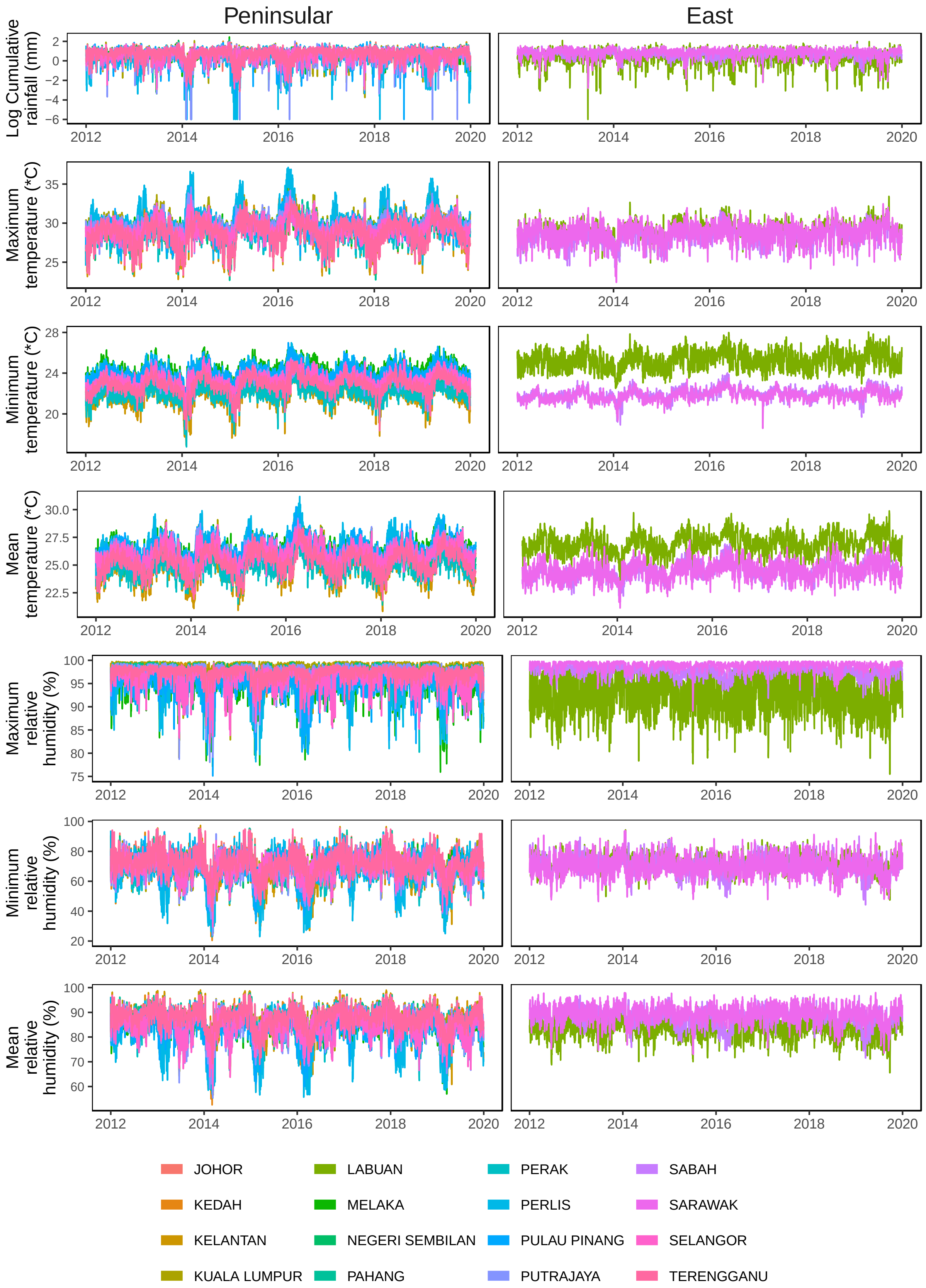

Figure S3. Meteorological variables. Meteorological variables for each day in 2012-2019 coloured for each state in Malaysia, arranged with states in Peninsular Malaysia in the left-hand panels and states in East Malaysia in the right-hand panels.
