# Supplementary material for: Spatio-temporal dynamics of hand, foot and mouth disease in Malaysia, 2009–2019
Source: PLoS Negl Trop Dis. 2025 Jun 9;19(6):e0013174. doi: 10.1371/journal.pntd.0013174 (PMC12180618; doi:10.1371/journal.pntd.0013174)
Supplement: S4 Fig — Districts are coloured according to their state. Population density is taken for 2019. The maximum number of consecutive months with zero reported cases is calculated over the period 2012–2019 for each district. (PDF) [file pntd.0013174.s004.pdf]

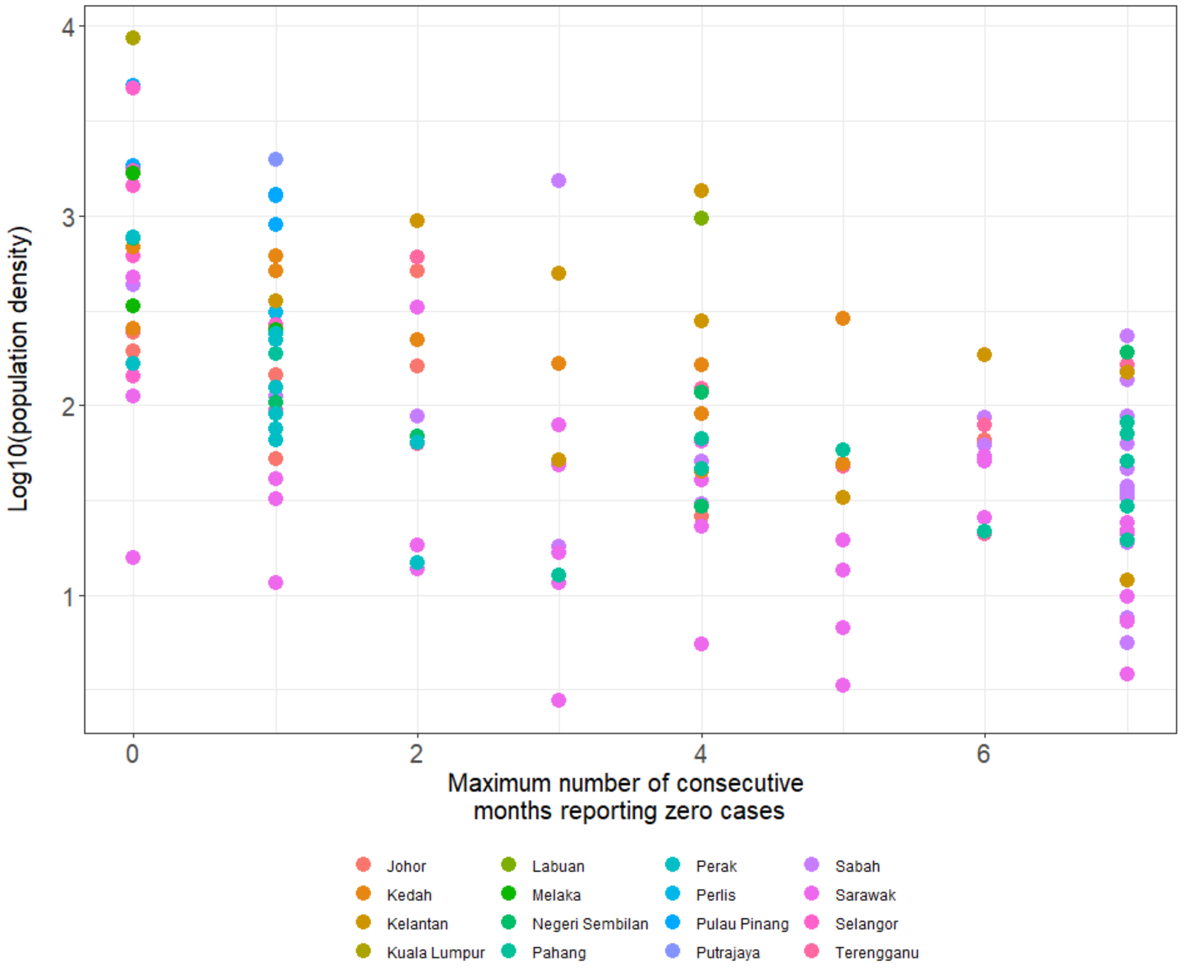

Figure S4. Relationship between district population density and maximum number of consecutive months reporting zero cases. Districts are coloured according to their state. Population density is taken for 2019. The maximum number of consecutive months with zero reported cases is calculated over the period 2012 to 2019 for each district.
