# Supplementary material for: Spatio-temporal dynamics of hand, foot and mouth disease in Malaysia, 2009–2019
Source: PLoS Negl Trop Dis. 2025 Jun 9;19(6):e0013174. doi: 10.1371/journal.pntd.0013174 (PMC12180618; doi:10.1371/journal.pntd.0013174)
Supplement: S6 Fig — Weekly distribution of annual cases within the year for all years between 2009 and 2019 in each state. (PDF) [file pntd.0013174.s006.pdf]

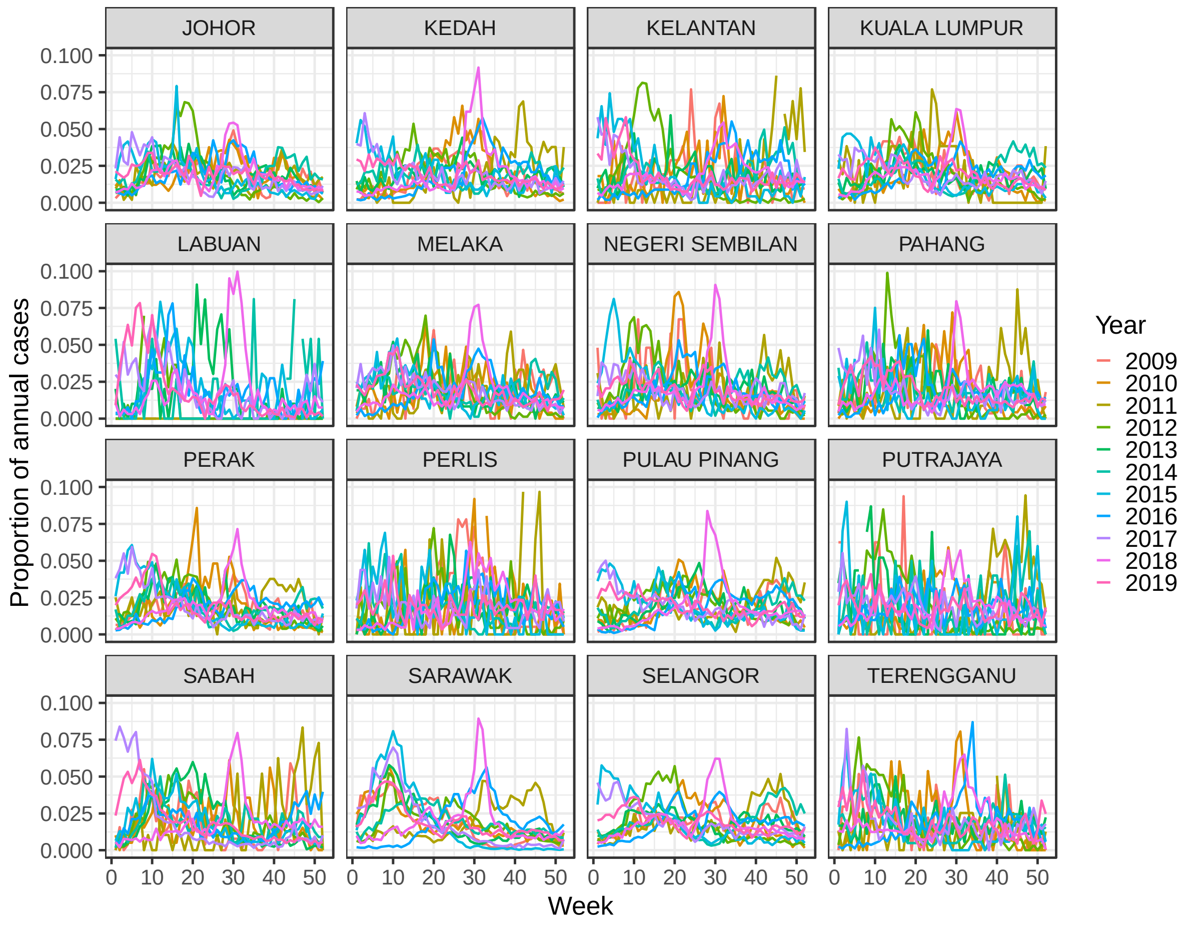

Figure S6. Distribution of cases within the year. Weekly distribution of annual cases within the year for all years between 2009 and 2019 in each state.
