# Supplementary material for: Spatio-temporal dynamics of hand, foot and mouth disease in Malaysia, 2009–2019
Source: PLoS Negl Trop Dis. 2025 Jun 9;19(6):e0013174. doi: 10.1371/journal.pntd.0013174 (PMC12180618; doi:10.1371/journal.pntd.0013174)
Supplement: S9 Fig — (Top) Daily incidence of HFMD cases, coloured by epidemic period (dark green) or not (light green), for Perak, Pulau Pinang and Selangor. (Bottom) Estimated median effective reproduction number with 50% and 95% credible intervals, and epidemic periods shown in dark green. (PDF) [file pntd.0013174.s009.pdf]

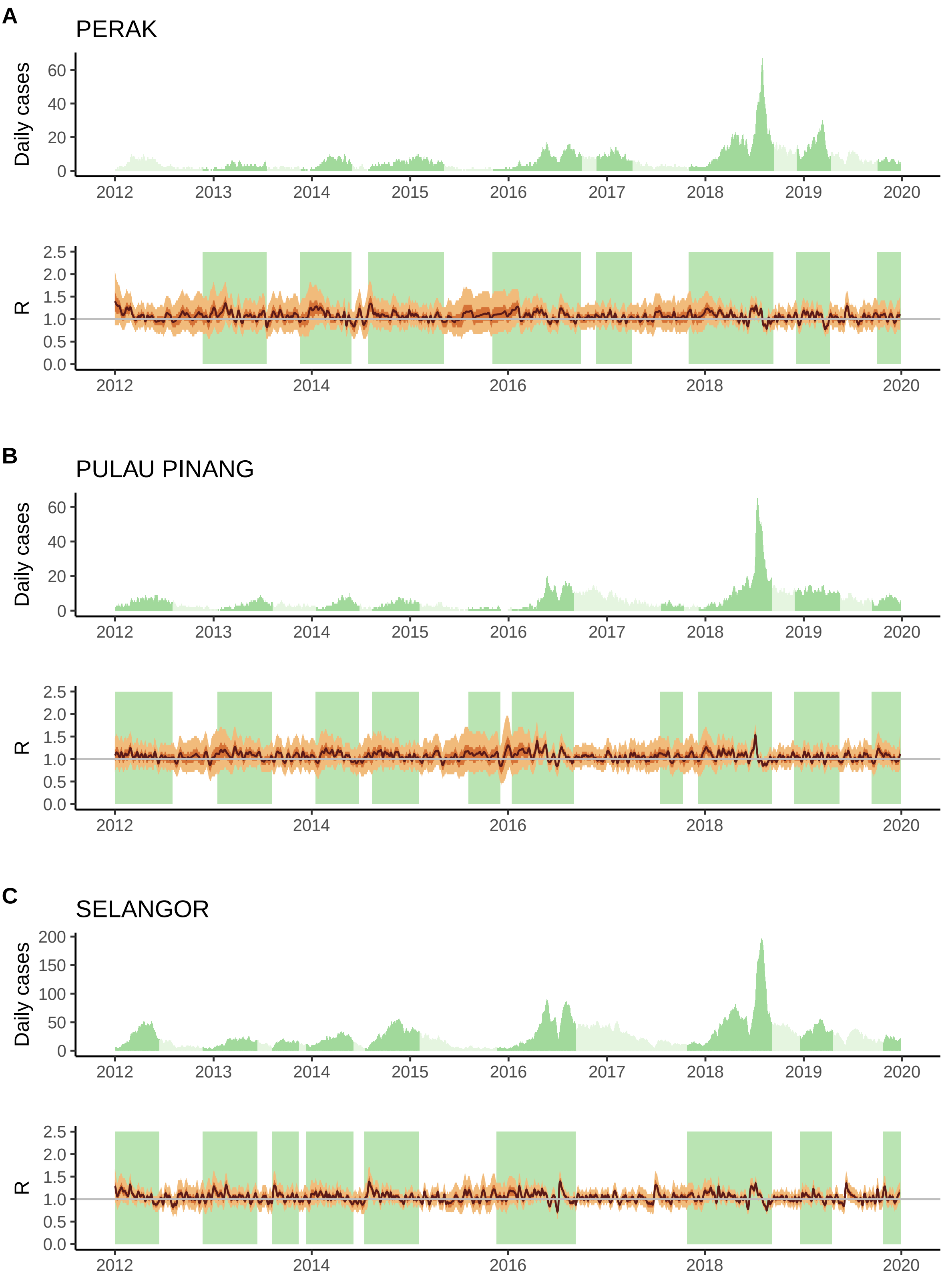

Figure S9. Incidence and effective reproduction number of HFMD per state. (Top) Daily incidence of HFMD cases, coloured by epidemic period (dark green) or not (light green), for Perak, Pulau Pinang and Selangor. (Bottom) Estimated median effective reproduction number with 50% and 95% credible intervals, and epidemic periods shown in dark green.
