# Supplementary material for: Spatio-temporal dynamics of hand, foot and mouth disease in Malaysia, 2009–2019
Source: PLoS Negl Trop Dis. 2025 Jun 9;19(6):e0013174. doi: 10.1371/journal.pntd.0013174 (PMC12180618; doi:10.1371/journal.pntd.0013174)
Supplement: S11 Fig — The dashed black vertical line indicates Rt = 1. (PDF) [file pntd.0013174.s011.pdf]

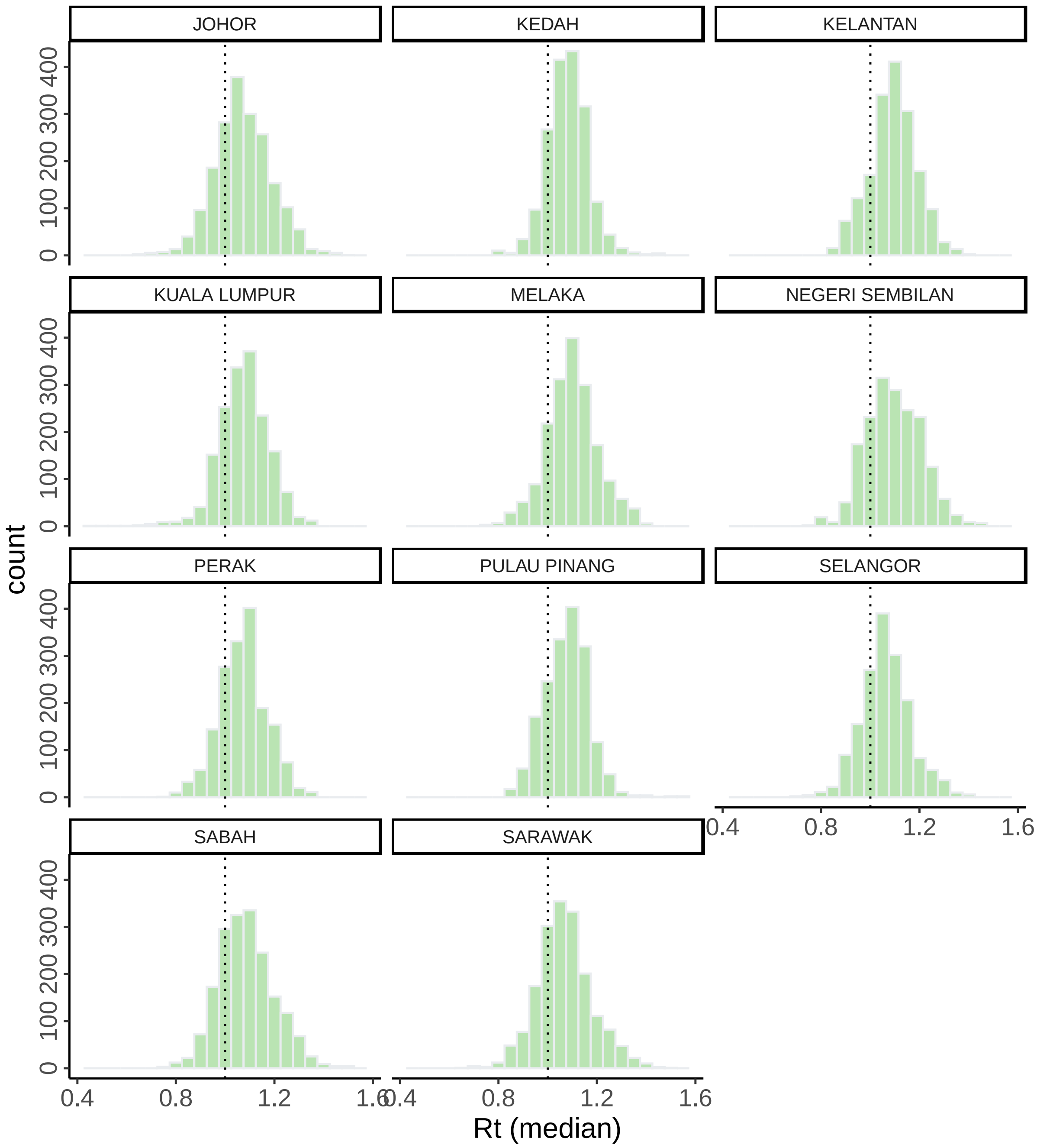

Figure S11. Distribution of daily median estimates of the effective reproduction number during epidemic periods in each state considered in the regression analysis. The dashed black vertical line indicates Rt=1.
