# Supplementary material for: Spatio-temporal dynamics of hand, foot and mouth disease in Malaysia, 2009–2019
Source: PLoS Negl Trop Dis. 2025 Jun 9;19(6):e0013174. doi: 10.1371/journal.pntd.0013174 (PMC12180618; doi:10.1371/journal.pntd.0013174)
Supplement: S12 Fig — Meteorological and school holiday variables at different lags (day 0–14, on the y-axis), and the proportion of EV-A71 positive samples relative to EV-A71 and/or CVA16 positive samples. The direction of the association is shown in blue for positive (beta>0) and in red for negative (beta<0) associations, and zero (no effect) is shown as a dotted vertical line. (PDF) [file pntd.0013174.s012.pdf]

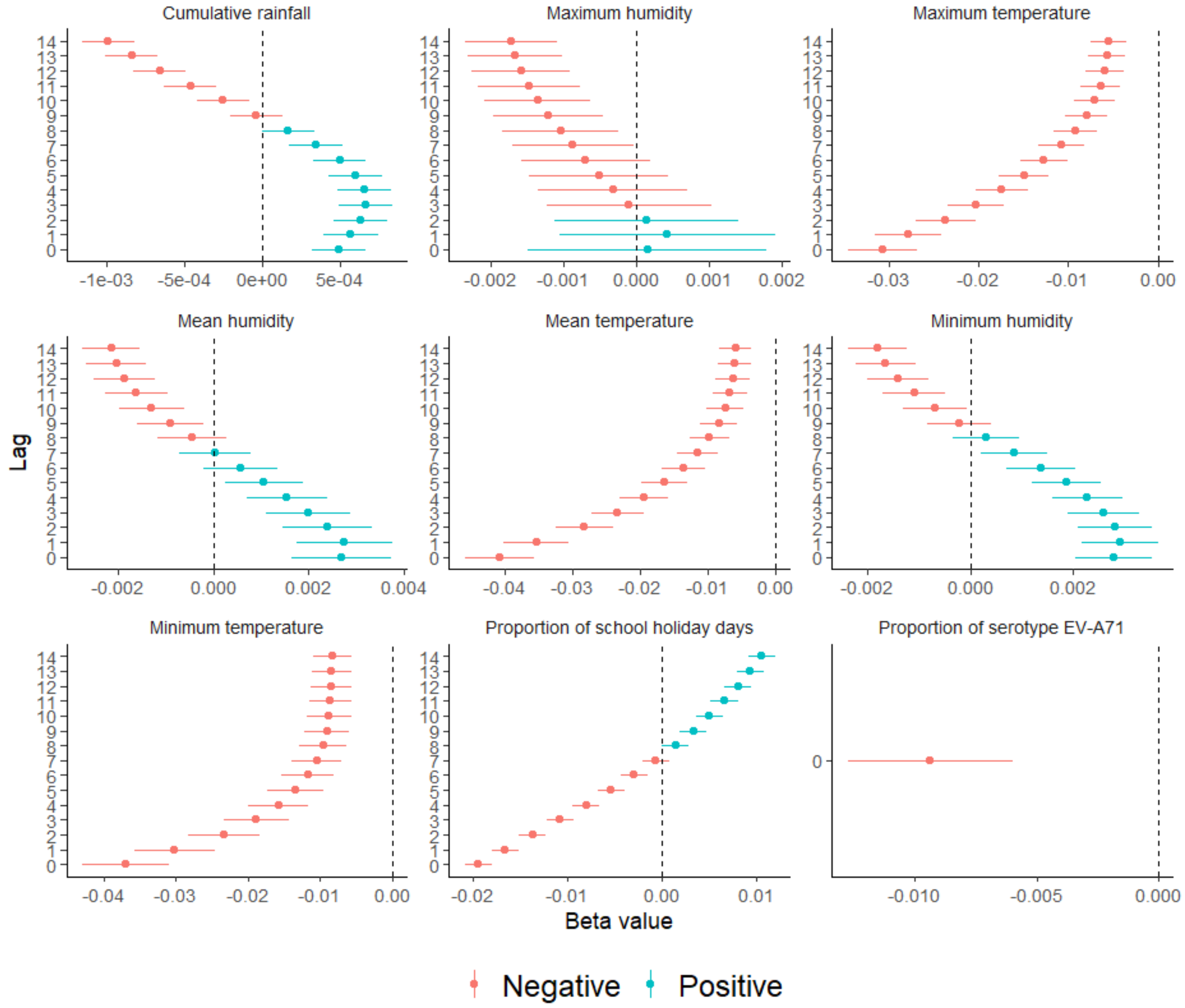

Figure S12. Estimated regression coefficients (beta value) for each fixed effect variable in the univariable models. Meteorological and school holiday variables at different lags (day 0 to 14, on the y-axis), and the proportion of EV-A71 positive samples relative to EV-A71 and/or CVA16 positive samples. The direction of the association is shown in blue for positive (beta>0) and in red for negative (beta<0) associations, and zero (no effect) is shown as a dotted vertical line.
