# Supplementary material for: Spatio-temporal dynamics of hand, foot and mouth disease in Malaysia, 2009–2019
Source: PLoS Negl Trop Dis. 2025 Jun 9;19(6):e0013174. doi: 10.1371/journal.pntd.0013174 (PMC12180618; doi:10.1371/journal.pntd.0013174)
Supplement: S13 Fig — Each variable included in the final model was removed, and the Watanabe-Akaike Information Criterion score (WAIC) and the Deviance Information Criterion score (DIC) of the resulting model is shown in red and blue respectively, with the best fitting and final model at the top, with the lowest WAIC and DIC. (PDF) [file pntd.0013174.s013.pdf]

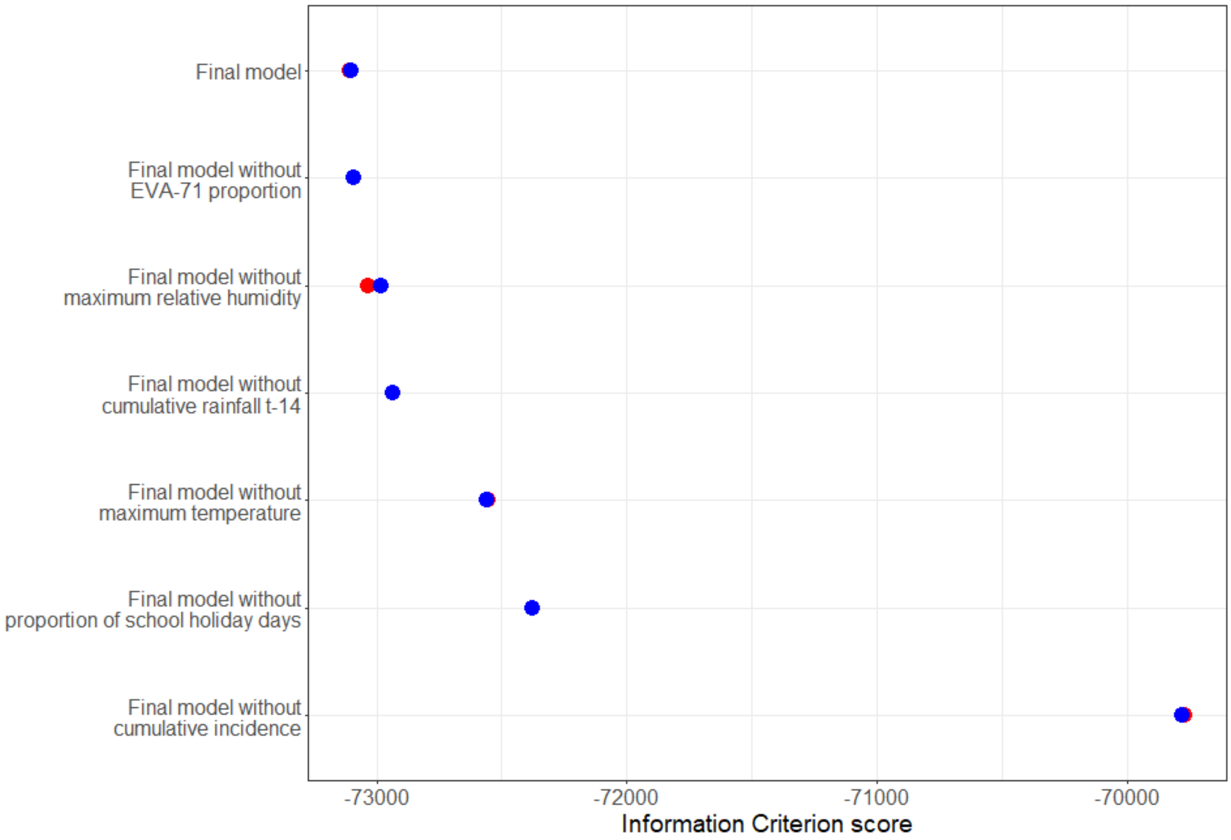

Figure S13. Relative effect of each individual variable included in the final model. Each variable included in the final model was removed, and the Watanabe-Akaike Information Criterion score (WAIC) and the Deviance Information Criterion score (DIC) of the resulting model is shown in red and blue respectively, with the best fitting and final model at the top, with the lowest WAIC and DIC.
