# Supplementary material for: Spatio-temporal dynamics of hand, foot and mouth disease in Malaysia, 2009–2019
Source: PLoS Negl Trop Dis. 2025 Jun 9;19(6):e0013174. doi: 10.1371/journal.pntd.0013174 (PMC12180618; doi:10.1371/journal.pntd.0013174)
Supplement: S15 Fig — Median estimates of the effective reproduction number during the epidemic periods obtained with EpiFilter are shown in grey, for Kuala Lumpur, Melaka and Negeri Sembilan. The estimates obtained with the final mixed-effects regression model are shown in red (median) and pink (95% CrI). We sampled 1000 times from the final model and extracted the 0.50, 0.025, and 0.975 quantiles to estimate the median Rt and the associated 95% CrIs. (PDF) [file pntd.0013174.s015.pdf]

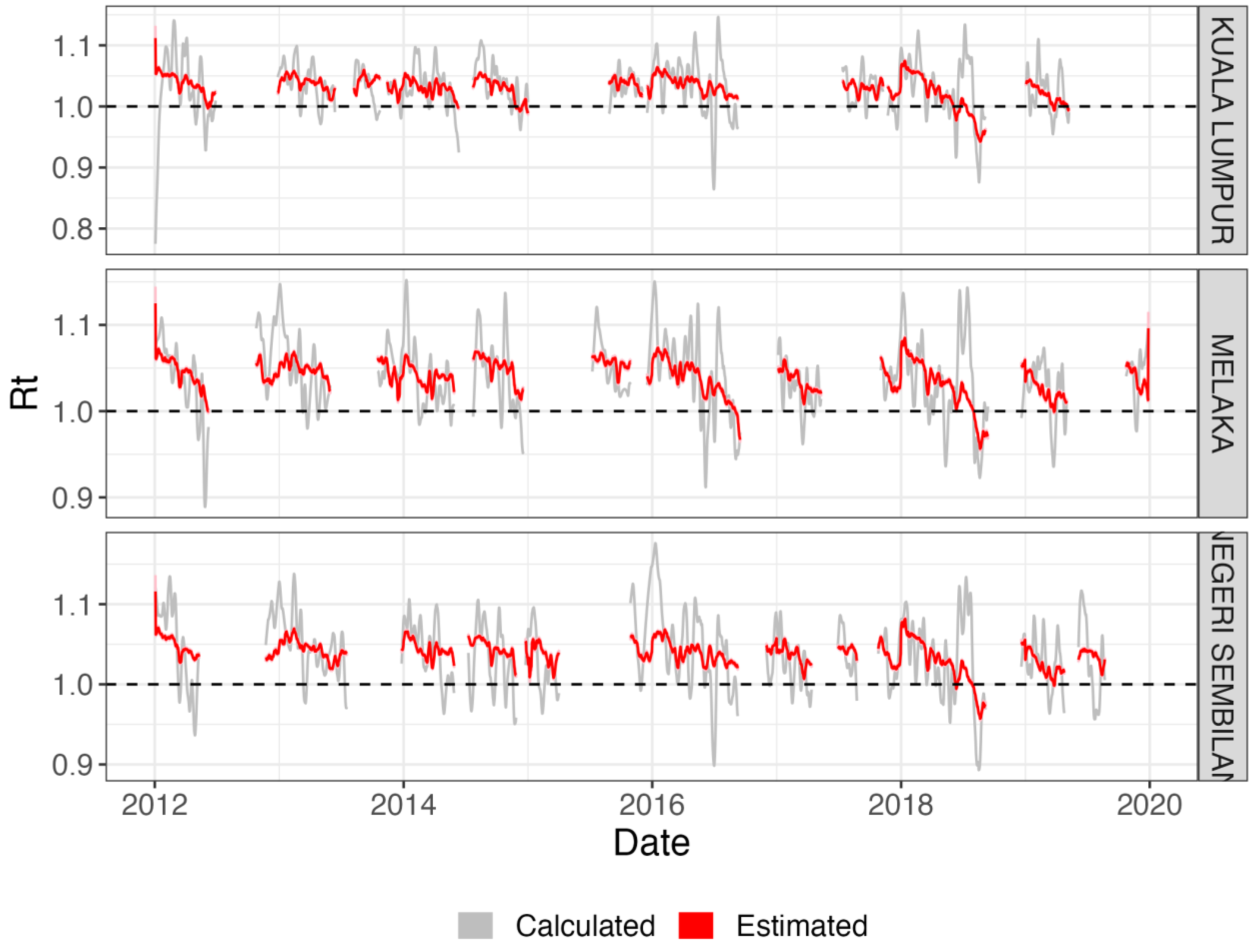

Figure S15. Model fit to data. Median estimates of the effective reproduction number during the epidemic periods obtained with EpiFilter are shown in grey, for Kuala Lumpur, Melaka and Negeri Sembilan. The estimates obtained with the final mixed-effects regression model are shown in red (median) and pink (95% CrI). We sampled 1000 times from the final model and extracted the 0.50, 0.025, and 0.975 quantiles to estimate the median Rt and the associated 95% CrIs.
