# Supplementary material for: Spatio-temporal dynamics of hand, foot and mouth disease in Malaysia, 2009–2019
Source: PLoS Negl Trop Dis. 2025 Jun 9;19(6):e0013174. doi: 10.1371/journal.pntd.0013174 (PMC12180618; doi:10.1371/journal.pntd.0013174)
Supplement: S20 Fig — Timeseries of the residuals (i.e., difference between observed and mean predicted values) of the final model of log(Rt) for Kuala Lumpur, Melaka and Negeri Sembilan. (PDF) [file pntd.0013174.s020.pdf]

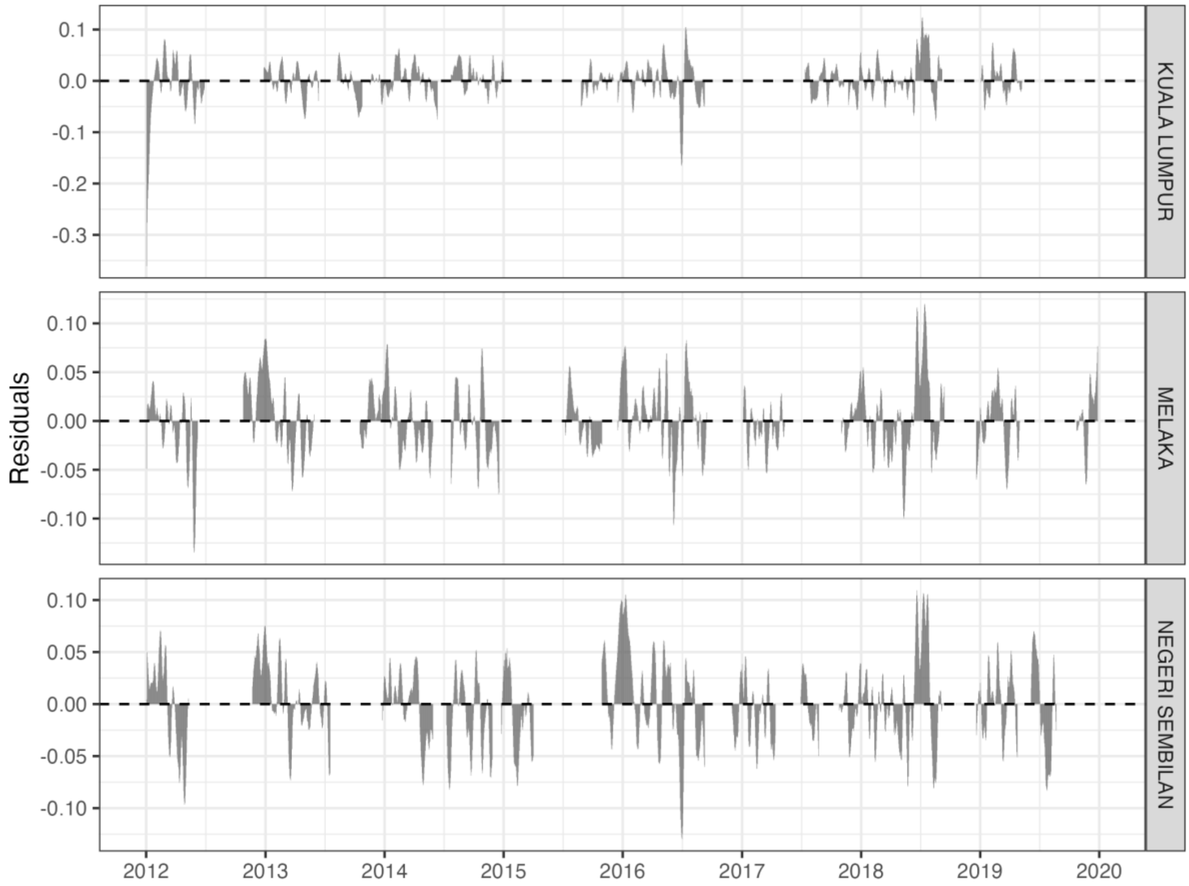

Figure S20. Time-series of the residuals. Time-series of the residuals (i.e. difference between observed and mean predicted values) of the final model of log(Rt) for Kuala Lumpur, Melaka and Negeri Sembilan.
