# Supplementary material for: Spatio-temporal dynamics of hand, foot and mouth disease in Malaysia, 2009–2019
Source: PLoS Negl Trop Dis. 2025 Jun 9;19(6):e0013174. doi: 10.1371/journal.pntd.0013174 (PMC12180618; doi:10.1371/journal.pntd.0013174)
Supplement: S23 Fig — (A) The random effect on year was modelled as a random walk of order 1 for each region, Peninsular and East Malaysia. (B) The random effect per state was modelled using an independent and identically distributed model. The base layer of the map was sourced from GADM (https://gadm.org/download_country.html and https://gadm.org/license.html). (PDF) [file pntd.0013174.s023.pdf]

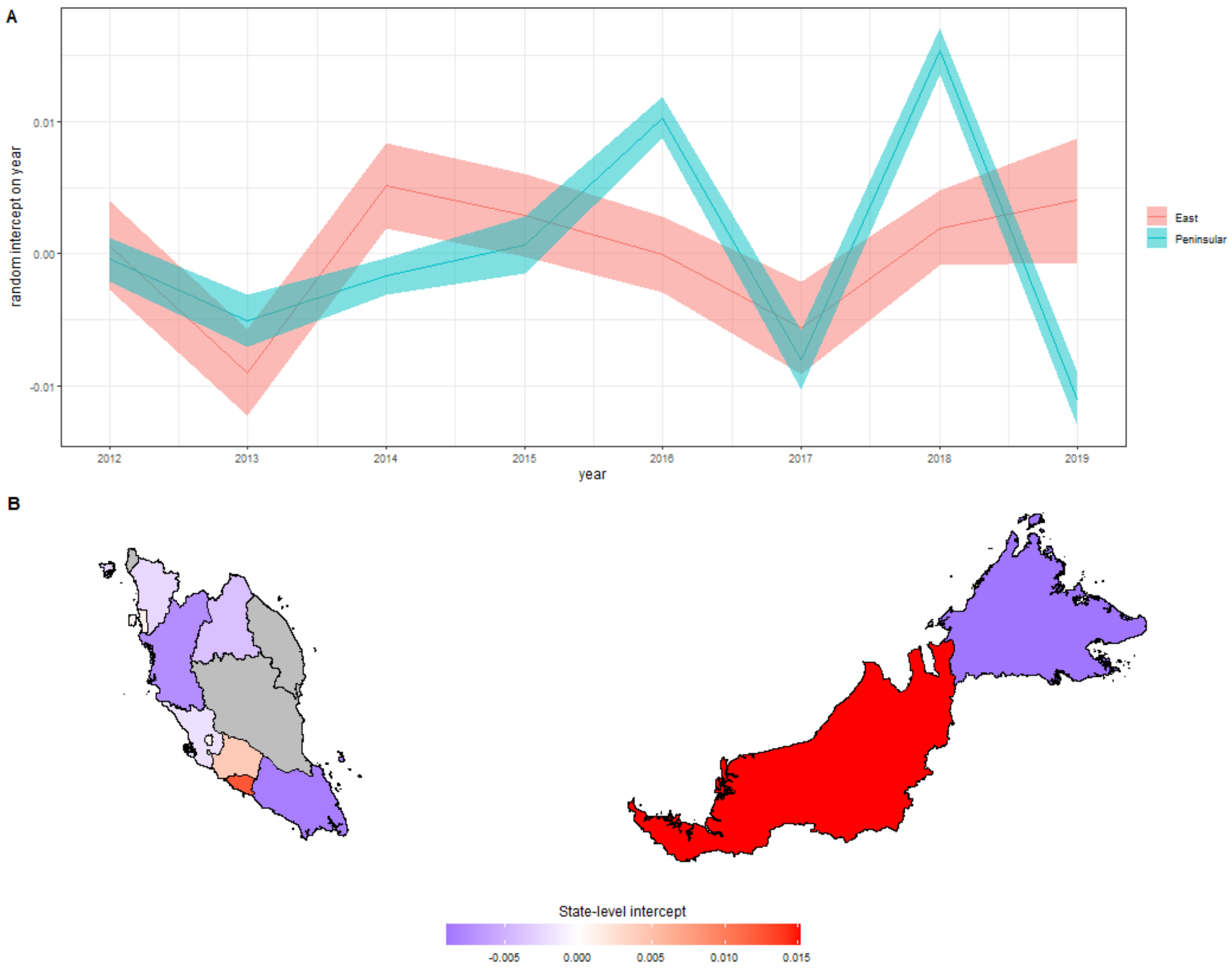

Figure S23. Spatial and temporal random effects in the final model. (A) The random effect on year was modelled as a random walk of order 1 for each region, Peninsular and East Malaysia. (B) The random effect per state was modelled using an independent and identically distributed model.
