# Supplementary material for: Spatio-temporal dynamics of hand, foot and mouth disease in Malaysia, 2009–2019
Source: PLoS Negl Trop Dis. 2025 Jun 9;19(6):e0013174. doi: 10.1371/journal.pntd.0013174 (PMC12180618; doi:10.1371/journal.pntd.0013174)
Supplement: S24 Fig — Regression coefficients for each percentile of the meteorological variables included as random effect categorical variables in univariable models. The meteorological variables were calculated at time t (lag = 0 days). (PDF) [file pntd.0013174.s024.pdf]

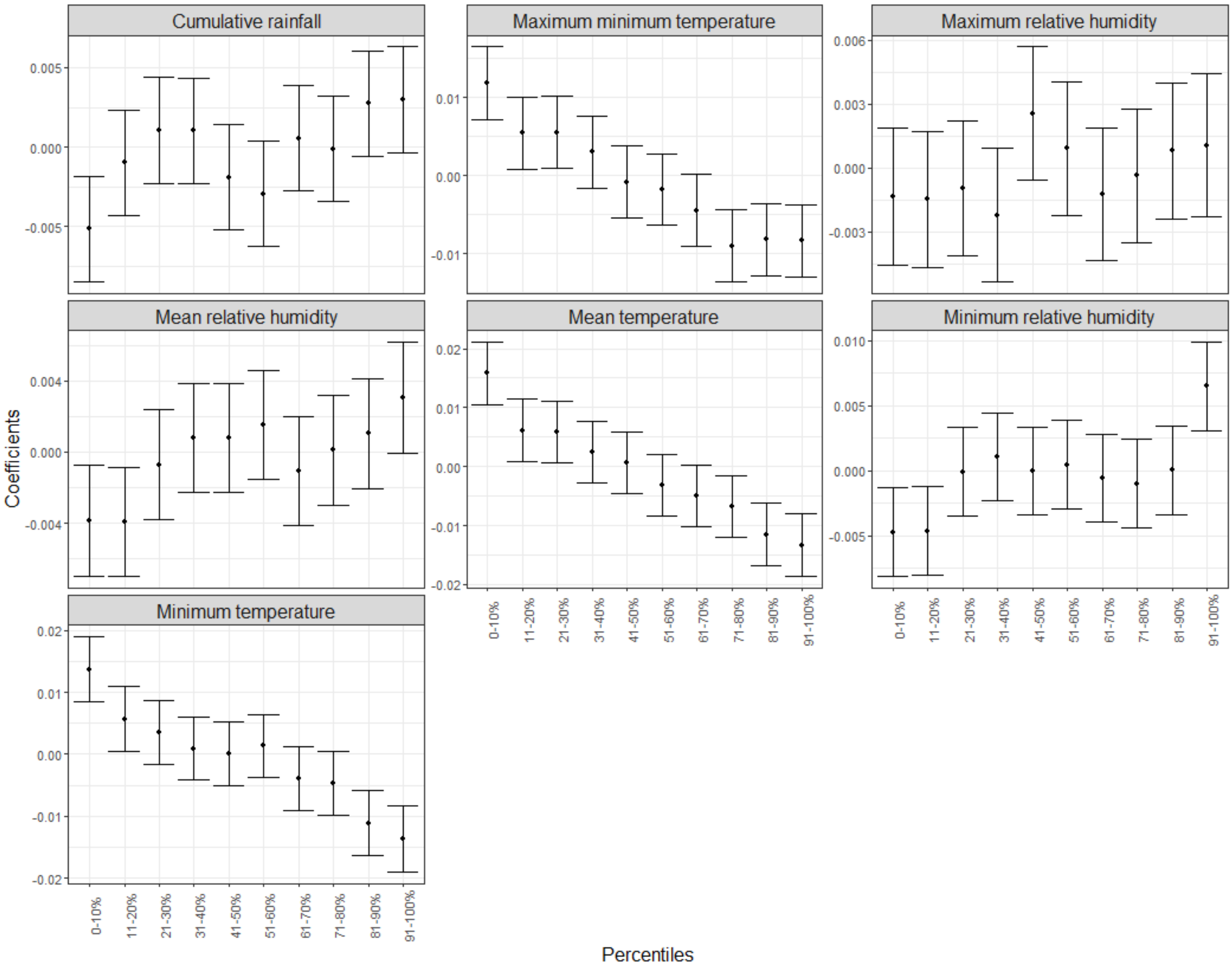

Figure S24. Sensitivity analysis testing non-linear relationships between meteorological variables and log(Rt). Regression coefficients for each percentile of the meteorological variables included as random effect categorical variables in univariable models. The meteorological variables were calculated at time t (lag = 0 days).
