# Supplementary material for: Spatio-temporal dynamics of hand, foot and mouth disease in Malaysia, 2009–2019
Source: PLoS Negl Trop Dis. 2025 Jun 9;19(6):e0013174. doi: 10.1371/journal.pntd.0013174 (PMC12180618; doi:10.1371/journal.pntd.0013174)
Supplement: S1 Table — We lagged the calculated proportion of school holiday days and meteorological variables each day between t and t-14 (n = 15 lags tested in the models). (DOCX) [file pntd.0013174.s025.docx]

**S1 Table. Summary of the variables tested in the mixed-effects regression models.** We lagged the calculated proportion of school holiday days and meteorological variables each day between t and t-14 (n=15 lags tested in the models).

| **Variable** | **Description** | **Spatial resolution** | **Temporal grain** | **Lags** | **Source** |
| --- | --- | --- | --- | --- | --- |
| **prop_hols** | Proportion of school holiday days (weekly sum of days of holiday divided by 7, centred around day t: t-3 to t+3) | State | Daily for 2012-2019 | t, t-1, t-2, t-3, t-4, t-5, t-6, t-7, t-8, t-9, t-10, t-11, t-12, t-13, t-14 | Ministry of Education, Malaysia https://www.moe.gov.my/ |
| **EVA -71** | EV-A71 monthly proportion (EV-A71 cases divided by the total number of serotyped EV-A71 and CVA16 cases) | National | Weekly for 2012 and 2014, 2015, 2016, 2017 and 2018 | N/A | Ministry of Health, Malaysia |
| **mean_hum** | Mean relative humidity % (weekly average of daily values centred around day t: t-3 to t+3) | State | Daily for 2012-2019 | t, t-1, t-2, t-3, t-4, t-5, t-6, t-7 t-8, t-9, t-10, t-11, t-12, t-13, t-14 | Copernicus Climate Change Service (C3S) |
| **min_hum** | Minimum relative humidity % (weekly average of daily values centred around day t: t-3 to t+3) | State | Daily for 2012-2019 | t, t-1, t-2, t-3, t-4, t-5, t-6, t-7 t-8, t-9, t-10, t-11, t-12, t-13, t-14 | Copernicus Climate Change Service (C3S) |
| **max_hum** | Maximum relative humidity % (weekly average of daily values centred around day t: t-3 to t+3) | State | Daily for 2012-2019 | t, t-1, t-2, t-3, t-4, t-5, t-6, t-7 t-8, t-9, t-10, t-11, t-12, t-13, t-14 | Copernicus Climate Change Service (C3S) |
| **mean_temp** | Mean temperature in degrees Celcius (weekly average of daily values centred around day t: t-3 to t+3) | State | Daily for 2012-2019 | t, t-1, t-2, t-3, t-4, t-5, t-6, t-7 t-8, t-9, t-10, t-11, t-12, t-13, t-14 | Copernicus Climate Change Service (C3S) |
| **min_temp** | Minimum temperature in degrees Celcius (weekly average of daily values centred around day t: t-3 to t+3) | State | Daily for 2012-2019 | t, t-1, t-2, t-3, t-4, t-5, t-6, t-7 t-8, t-9, t-10, t-11, t-12, t-13, t-14 | Copernicus Climate Change Service (C3S) |
| **max_temp** | Maximum temperature in degrees Celcius (weekly average of daily values centred around day t: t-3 to t+3) | State | Daily for 2012-2019 | t, t-1, t-2, t-3, t-4, t-5, t-6, t-7 t-8, t-9, t-10, t-11, t-12, t-13, t-14 | Copernicus Climate Change Service (C3S) |
| **cum_rf** | Cumulative rainfall in mm (weekly sum of daily values centred around day t: t-3 to t+3) | State | Daily for 2012-2019 | t, t-1, t-2, t-3, t-4, t-5, t-6, t-7 t-8, t-9, t-10, t-11, t-12, t-13, t-14 | Copernicus Climate Change Service (C3S) |
