# Supplementary material for: Spatio-temporal dynamics of hand, foot and mouth disease in Malaysia, 2009–2019
Source: PLoS Negl Trop Dis. 2025 Jun 9;19(6):e0013174. doi: 10.1371/journal.pntd.0013174 (PMC12180618; doi:10.1371/journal.pntd.0013174)
Supplement: S2 Table — (DOCX) [file pntd.0013174.s026.docx]

**S2 Table. Univariable model variables arranged from top to bottom by the best WAIC score.**

| **Order WAIC** | **Order DIC** | **Variable** | **Lag** | **WAIC** | | **DIC** | **Regression coefficient, β (95% CrI)** |
| --- | --- | --- | --- | --- | --- | --- | --- |
| 1 | 1 | Proportion of school holiday days | 0 | | -72502 | -72502.6 | -0.0194 (-0.0208, -0.018) |
| 2 | 2 | Proportion of school holiday days | 1 | | -72304.4 | -72305 | -0.0165 (-0.0179, -0.0151) |
| 3 | 3 | Proportion of school holiday days | 2 | | -72138.7 | -72139.3 | -0.0136 (-0.0151, -0.0122) |
| 4 | 5 | Mean temperature | 0 | | -72055.5 | -72028.5 | -0.0408 (-0.046, -0.0357) |
| 5 | 4 | Maximum temperature | 0 | | -72051.8 | -72029.7 | -0.0307 (-0.0345, -0.0268) |
| 6 | 7 | Maximum temperature | 1 | | -72030.2 | -72007.2 | -0.0278 (-0.0315, -0.0242) |
| 7 | 9 | Mean temperature | 1 | | -72021.2 | -71992.7 | -0.0354 (-0.0402, -0.0305) |
| 8 | 6 | Proportion of school holiday days | 3 | | -72006.6 | -72007.3 | -0.0108 (-0.0122, -0.00935) |
| 9 | 8 | Proportion of school holiday days | 14 | | -71998.4 | -71999.1 | 0.0105 (0.00909, 0.0119) |
| 10 | 10 | Maximum temperature | 2 | | -71987.6 | -71976.8 | -0.0236 (-0.027, -0.0202) |
| 11 | 12 | Mean temperature | 2 | | -71964.6 | -71953.1 | -0.0283 (-0.0326, -0.024) |
| 12 | 13 | Maximum temperature | 3 | | -71955.2 | -71949.5 | -0.0202 (-0.0234, -0.0171) |
| 13 | 11 | Proportion of school holiday days | 13 | | -71953.4 | -71954.2 | 0.00932 (0.0079, 0.0107) |
| 14 | 14 | Minimum temperature | 0 | | -71949.2 | -71933 | -0.037 (-0.0431, -0.0309) |
| 15 | 16 | Mean temperature | 3 | | -71927.8 | -71923.2 | -0.0233 (-0.0273, -0.0194) |
| 16 | 15 | Maximum temperature | 4 | | -71925.9 | -71923.9 | -0.0174 (-0.0203, -0.0145) |
| 17 | 17 | Cumulative rainfall | 14 | | -71919.5 | -71920.4 | -0.000991 (-0.00116, -0.000822) |
| 18 | 18 | Proportion of school holiday days | 12 | | -71910.1 | -71910.9 | 0.00801 (0.00659, 0.00943) |
| 19 | 19 | Proportion of school holiday days | 4 | | -71908.8 | -71909.6 | -0.00801 (-0.00944, -0.00659) |
| 20 | 21 | Minimum temperature | 1 | | -71907.2 | -71899.5 | -0.0302 (-0.0358, -0.0246) |
| 21 | 22 | Mean temperature | 4 | | -71898.7 | -71898.1 | -0.0195 (-0.0231, -0.0158) |
| 22 | 20 | Maximum temperature | 5 | | -71898.4 | -71899.5 | -0.0149 (-0.0177, -0.0121) |
| 23 | 23 | Cumulative rainfall | 13 | | -71882.5 | -71883.3 | -0.00084 (-0.00101, -0.000671) |
| 24 | 26 | Minimum temperature | 2 | | -71874 | -71872.9 | -0.0233 (-0.0283, -0.0183) |
| 25 | 24 | Maximum temperature | 6 | | -71873.7 | -71877.4 | -0.0127 (-0.0153, -0.01) |
| 26 | 25 | Mean temperature | 5 | | -71873.5 | -71876.2 | -0.0163 (-0.0197, -0.0129) |
| 27 | 27 | Proportion of school holiday days | 11 | | -71870.2 | -71871 | 0.00658 (0.00516, 0.008) |
| 28 | 28 | Maximum temperature | 7 | | -71855.3 | -71858.7 | -0.0108 (-0.0133, -0.00825) |
| 29 | 29 | Mean temperature | 6 | | -71853.3 | -71857.4 | -0.0136 (-0.0169, -0.0104) |
| 30 | 30 | Minimum temperature | 3 | | -71852.8 | -71855.8 | -0.0189 (-0.0234, -0.0144) |
| 31 | 31 | Minimum humidity | 1 | | -71848.9 | -71850.1 | 0.00293 (0.00219, 0.00366) |
| 32 | 32 | Minimum humidity | 2 | | -71847.1 | -71848.4 | 0.00282 (0.0021, 0.00354) |
| 33 | 33 | Cumulative rainfall | 12 | | -71846.3 | -71847.1 | -0.00066 (-0.00083, -0.000491) |
| 34 | 34 | Cumulative rainfall | 3 | | -71844.5 | -71845.1 | 0.000658 (0.000487, 0.00083) |
| 35 | 35 | Cumulative rainfall | 4 | | -71843.5 | -71844.2 | 0.000651 (0.00048, 0.000823) |
| 36 | 38 | Proportion of school holiday days | 5 | | -71842.6 | -71843.4 | -0.00539 (-0.00681, -0.00396) |
| 37 | 39 | Minimum humidity | 0 | | -71842.5 | -71843.4 | 0.0028 (0.00205, 0.00355) |
| 38 | 36 | Maximum temperature | 8 | | -71840.8 | -71843.9 | -0.00918 (-0.0116, -0.00677) |
| 39 | 41 | Minimum humidity | 3 | | -71840.6 | -71842.1 | 0.0026 (0.0019, 0.0033) |
| 40 | 37 | Minimum temperature | 4 | | -71839.1 | -71843.6 | -0.0157 (-0.0199, -0.0116) |
| 41 | 42 | Cumulative rainfall | 2 | | -71838.5 | -71839.2 | 0.000625 (0.000453, 0.000798) |
| 42 | 40 | Mean temperature | 7 | | -71838.4 | -71842.1 | -0.0114 (-0.0145, -0.00839) |
| 43 | 43 | Proportion of school holiday days | 10 | | -71835.5 | -71836.3 | 0.00501 (0.00359, 0.00643) |
| 44 | 44 | Cumulative rainfall | 5 | | -71834.7 | -71835.4 | 0.000596 (0.000425, 0.000767) |
| 45 | 45 | Mean humidity | 14 | | -71833.3 | -71835.2 | -0.00215 (-0.00276, -0.00153) |
| 46 | 47 | Maximum temperature | 9 | | -71830.3 | -71833.3 | -0.00795 (-0.0103, -0.00562) |
| 47 | 46 | Minimum temperature | 5 | | -71830.3 | -71834.5 | -0.0134 (-0.0173, -0.00956) |
| 48 | 48 | Minimum humidity | 4 | | -71830 | -71831.5 | 0.00228 (0.00159, 0.00297) |
| 49 | 50 | Cumulative rainfall | 1 | | -71828.8 | -71829.4 | 0.000565 (0.000392, 0.000739) |
| 50 | 49 | Mean temperature | 8 | | -71827.1 | -71830.5 | -0.00969 (-0.0126, -0.00677) |
| 51 | 51 | Mean humidity | 13 | | -71826.8 | -71828.8 | -0.00204 (-0.00267, -0.00141) |
| 52 | 52 | Minimum temperature | 6 | | -71823.8 | -71827.8 | -0.0117 (-0.0153, -0.00805) |
| 53 | 55 | Minimum humidity | 14 | | -71823.7 | -71825.1 | -0.00181 (-0.0024, -0.00123) |
| 54 | 53 | Maximum temperature | 10 | | -71823.3 | -71826 | -0.00703 (-0.00928, -0.00478) |
| 55 | 54 | Minimum temperature | 14 | | -71822.9 | -71825.1 | -0.00828 (-0.0109, -0.00561) |
| 56 | 56 | Minimum temperature | 13 | | -71821.5 | -71823.9 | -0.00837 (-0.0111, -0.00563) |
| 57 | 62 | Cumulative rainfall | 6 | | -71820.1 | -71820.8 | 0.000494 (0.000324, 0.000665) |
| 58 | 59 | Minimum temperature | 12 | | -71819.8 | -71822.3 | -0.00845 (-0.0113, -0.00562) |
| 59 | 57 | Minimum temperature | 7 | | -71819.6 | -71823.2 | -0.0104 (-0.0139, -0.00697) |
| 60 | 58 | Mean temperature | 9 | | -71819.2 | -71822.4 | -0.00835 (-0.0111, -0.00555) |
| 61 | 60 | Maximum temperature | 11 | | -71818.8 | -71821.4 | -0.00637 (-0.00854, -0.00419) |
| 62 | 63 | Mean humidity | 12 | | -71818.5 | -71820.6 | -0.00186 (-0.00251, -0.00122) |
| 63 | 61 | Minimum temperature | 11 | | -71818.3 | -71821 | -0.00855 (-0.0115, -0.00562) |
| 64 | 67 | Proportion of serotype EV-A71 | NA | | -71818 | -71818.7 | -0.00938 (-0.0128, -0.00598) |
| 65 | 70 | Cumulative rainfall | 0 | | -71817.8 | -71818.5 | 0.000488 (0.000313, 0.000662) |
| 66 | 64 | Minimum temperature | 8 | | -71817.3 | -71820.5 | -0.00958 (-0.0129, -0.00628) |
| 67 | 65 | Minimum temperature | 10 | | -71817.1 | -71820 | -0.00874 (-0.0118, -0.0057) |
| 68 | 68 | Minimum humidity | 5 | | -71817.1 | -71818.7 | 0.00187 (0.00119, 0.00254) |
| 69 | 75 | Cumulative rainfall | 11 | | -71816.8 | -71817.5 | -0.000465 (-0.000635, -0.000295) |
| 70 | 66 | Minimum temperature | 9 | | -71816.7 | -71819.8 | -0.00905 (-0.0122, -0.00589) |
| 71 | 71 | Minimum humidity | 13 | | -71816.7 | -71818.1 | -0.00165 (-0.00224, -0.00106) |
| 72 | 69 | Maximum temperature | 12 | | -71816.3 | -71818.6 | -0.00593 (-0.00805, -0.00382) |
| 73 | 72 | Maximum temperature | 14 | | -71815.6 | -71817.7 | -0.00553 (-0.00754, -0.00353) |
| 74 | 73 | Maximum temperature | 13 | | -71815.5 | -71817.7 | -0.00568 (-0.00773, -0.00362) |
| 75 | 74 | Mean humidity | 1 | | -71815.2 | -71817.7 | 0.00275 (0.00175, 0.00375) |
| 76 | 77 | Maximum humidity | 14 | | -71814.4 | -71816.7 | -0.00172 (-0.00235, -0.00108) |
| 77 | 76 | Mean temperature | 10 | | -71814.1 | -71817 | -0.00737 (-0.0101, -0.00468) |
| 78 | 78 | Mean humidity | 0 | | -71812.3 | -71814 | 0.00268 (0.00164, 0.00372) |
| 79 | 79 | Mean humidity | 2 | | -71811 | -71813.8 | 0.0024 (0.00146, 0.00334) |
| 80 | 80 | Mean temperature | 1 | | -71810.9 | -71813.6 | -0.00668 (-0.00928, -0.00408) |
| 81 | 81 | Maximum humidity | 13 | | -71810.9 | -71813.4 | -0.00166 (-0.00231, -0.00101) |
| 82 | 83 | Mean humidity | 11 | | -71809.2 | -71811.4 | -0.00161 (-0.00227, -0.000953) |
| 83 | 82 | Mean temperature | 12 | | -71809.1 | -71811.7 | -0.00623 (-0.00874, -0.00372) |
| 84 | 84 | Mean temperature | 14 | | -71809.1 | -71811.2 | -0.00582 (-0.0082, -0.00344) |
| 85 | 85 | Mean temperature | 12 | | -71808.8 | -71811.2 | -0.00597 (-0.00841, -0.00353) |
| 86 | 88 | Proportion of school holiday days | 9 | | -71808.3 | -71809.1 | 0.00329 (0.00186, 0.00471) |
| 87 | 87 | Minimum humidity | 12 | | -71808.2 | -71809.6 | -0.00142 (-0.00202, -0.000816) |
| 88 | 86 | Maximum humidity | 12 | | -71806.9 | -71809.6 | -0.00158 (-0.00225, -0.000906) |
| 89 | 89 | Mean humidity | 3 | | -71805.3 | -71808.2 | 0.002 (0.00111, 0.00289) |
| 90 | 90 | Minimum humidity | 6 | | -71804.2 | -71805.9 | 0.00138 (0.000718, 0.00204) |
| 91 | 92 | Proportion of school holiday days | 6 | | -71804.2 | -71805 | -0.00294 (-0.00437, -0.00151) |
| 92 | 93 | Cumulative rainfall | 7 | | -71803.7 | -71804.3 | 0.000344 (0.000173, 0.000515) |
| 93 | 91 | Maximum humidity | 11 | | -71802.8 | -71805.7 | -0.00148 (-0.00217, -0.000778) |
| 94 | 94 | Mean humidity | 10 | | -71800.2 | -71802.5 | -0.0013 (-0.00197, -0.000616) |
| 95 | 97 | Minimum humidity | 11 | | -71799.3 | -71800.7 | -0.0011 (-0.0017, -0.000486) |
| 96 | 95 | Maximum humidity | 10 | | -71798.8 | -71801.9 | -0.00135 (-0.00208, -0.000625) |
| 97 | 96 | Mean humidity | 4 | | -71798.7 | -71801.7 | 0.00155 (0.000704, 0.0024) |
| 98 | 99 | Cumulative rainfall | 10 | | -71796.6 | -71797.3 | -0.000253 (-0.000423, -0.0000828) |
| 99 | 98 | Maximum humidity | 9 | | -71795 | -71798.3 | -0.0012 (-0.00196, -0.000447) |
| 100 | 100 | Minimum humidity | 7 | | -71793.9 | -71795.5 | 0.00085 (0.0002, 0.0015) |
| 101 | 103 | Mean humidity | 9 | | -71792.5 | -71794.9 | -0.000897 (-0.0016, -0.000197) |
| 102 | 101 | Mean humidity | 5 | | -71792.5 | -71795.3 | 0.00106 (0.000253, 0.00187) |
| 103 | 104 | Minimum humidity | 10 | | -71791.8 | -71793.3 | -0.000696 (-0.00131, -0.0000773) |
| 104 | 102 | Maximum humidity | 8 | | -71791.7 | -71795.2 | -0.00104 (-0.00183, -0.000245) |
| 105 | 107 | Cumulative rainfall | 8 | | -71791.6 | -71792.1 | 0.000165 (-0.00000563, 0.000336) |
| 106 | 106 | Proportion of school holiday days | 8 | | -71791.5 | -71792.4 | 0.0014 (-0.0000263, 0.00282) |
| 107 | 105 | Maximum humidity | 7 | | -71789 | -71792.8 | -0.000868 (-0.0017, -0.0000334) |
| 108 | 113 | Proportion of school holiday days | 7 | | -71788.8 | -71789.6 | -0.000678 (-0.0021, 0.000748) |
| 109 | 117 | Cumulative rainfall | 9 | | -71788.3 | -71788.9 | -0.0000403 (-0.00021, 0.00013) |
| 110 | 112 | Minimum humidity | 8 | | -71788.1 | -71789.7 | 0.000306 (-0.000333, 0.000945) |
| 111 | 109 | Mean humidity | 6 | | -71788 | -71790.7 | 0.000561 (-0.000216, 0.00134) |
| 112 | 110 | Mean humidity | 8 | | -71787.6 | -71790.1 | -0.000445 (-0.00117, 0.000278) |
| 113 | 114 | Minimum humidity | 9 | | -71787.6 | -71789.1 | -0.000221 (-0.00085, 0.000407) |
| 114 | 108 | Maximum humidity | 6 | | -71787 | -71791 | -0.000696 (-0.00158, 0.000189) |
| 115 | 121 | Mean humidity | 7 | | -71786.1 | -71788.7 | 0.0000477 (-0.000701, 0.000796) |
| 116 | 111 | Maximum humidity | 5 | | -71785.4 | -71789.8 | -0.000514 (-0.00146, 0.000432) |
| 117 | 119 | Maximum humidity | 0 | | -71784.7 | -71788.7 | 0.000152 (-0.00148, 0.00179) |
| 118 | 115 | Maximum humidity | 4 | | -71784.4 | -71789.1 | -0.000318 (-0.00134, 0.000706) |
| 119 | 120 | Maximum humidity | 3 | | -71783.7 | -71788.7 | -0.0000989 (-0.00122, 0.00102) |
| 120 | 116 | Maximum humidity | 1 | | -71783.6 | -71789 | 0.000427 (-0.00105, 0.0019) |
| 121 | 118 | Maximum humidity | 2 | | -71783.5 | -71788.8 | 0.000144 (-0.00112, 0.00141) |
