# Supplementary material for: Spatio-temporal dynamics of hand, foot and mouth disease in Malaysia, 2009–2019
Source: PLoS Negl Trop Dis. 2025 Jun 9;19(6):e0013174. doi: 10.1371/journal.pntd.0013174 (PMC12180618; doi:10.1371/journal.pntd.0013174)
Supplement: S3 Table — The final model is shown in bold. (DOCX) [file pntd.0013174.s027.docx]

**S3 Table. Variable selection for the final multivariable model.** The final model is shown in bold.

| **Model building step** | **Model** | **WAIC** | **DIC** |
| --- | --- | --- | --- |
|  | Log(Rt) ~ Baseline | -71790 | -71791 |
| 1 | Log(Rt) ~ Baseline + prop_hols | -72502 | -72503 |
| 2 | Log(Rt) ~ Baseline + prop_hols + max_temp | -72894 | -72855 |
| 3 | Log(Rt) ~ Baseline + prop_hols + max_temp + cum_rf14 + | -73029 | -72979 |
| 4 | Log(Rt) ~ Baseline + prop_hols + max_temp + cum_rf14 + max_hum | -73092 | -73091 |
| **5** | **Log(Rt) ~ Baseline + prop_hols + max_temp + cum_rf14 + max_hum + EV-A71** | **-73105** | **-73104** |
| 6 | Log(Rt) ~ Baseline + prop_hols + max_temp + cum_rf14 + max_hum + EV-A71 + min_temp | -73105 | -73108 |
