# Supplementary material for: Spatio-temporal dynamics of hand, foot and mouth disease in Malaysia, 2009–2019
Source: PLoS Negl Trop Dis. 2025 Jun 9;19(6):e0013174. doi: 10.1371/journal.pntd.0013174 (PMC12180618; doi:10.1371/journal.pntd.0013174)
Supplement: Attachment — Submitted filename: point-by-point_response_final.docx [file pntd.0013174.s029.docx]

**Additional Editor Comments:**

The Authors are expected to address all the criticisms by all Reviewers. In particular, please consider or discuss alternative model specifications or model evaluation metrics, confirm the accessibility of the provided github repository (Reviewer #1), and consider using more recent data or genetic evolutionary analysis to enrich the analysis (Reviewer #2). In additional to these comments, please address:

1. Table 1. Cumulative incidence was defined from the start of an epidemic period up to day t. Could the authors indicate in at least one of the figures the starting time of the epidemic periods?

The epidemic periods are shown in green for all states in Supplementary Figures S7 to S10.

**Journal Requirements:**

1) Please provide an Author Summary. This should appear in your manuscript between the Abstract (if applicable) and the Introduction, and should be 150-200 words long. The aim should be to make your findings accessible to a wide audience that includes both scientists and non-scientists. Sample summaries can be found on our website under Submission Guidelines:
<https://journals.plos.org/plosntds/s/submission-guidelines#loc-parts-of-a-submission>

Author Summary now provided.

2) Please upload all main figures as separate Figure files in .tif or .eps format. For more information about how to convert and format your figure files please see our guidelines:
<https://journals.plos.org/plosntds/s/figures>

Figures converted to required format.

3) We have noticed that you have uploaded Supporting Information files, but you have not included a list of legends. Please add a full list of legends for your Supporting Information files after the references list.

We have added the full list of legends for the Supporting Information after the references in the main text, and changed their citation accordingly.

4) Some material included in your submission may be copyrighted. According to PLOSu2019s copyright policy, authors who use figures or other material (e.g., graphics, clipart, maps) from another author or copyright holder must demonstrate or obtain permission to publish this material under the Creative Commons Attribution 4.0 International (CC BY 4.0) License used by PLOS journals. Please closely review the details of PLOSu2019s copyright requirements here: PLOS Licenses and Copyright. If you need to request permissions from a copyright holder, you may use PLOS's

Copyright Content Permission form.
Please respond directly to this email and provide any known details concerning your material's license terms and permissions required for reuse, even if you have not yet obtained copyright permissions or are unsure of your material's copyright compatibility. Once you have responded and addressed all other outstanding technical requirements, you may resubmit your manuscript within Editorial Manager.

Potential Copyright Issues:

- Figures 4C, 5B, and S17-B; Please provide a direct link to the base layer of the map (i.e., the country or region border shape) and ensure this is also included in the figure legend; and provide a link to the terms of use / license information for the base layer image or shapefile. We cannot publish proprietary or copyrighted maps (e.g. Google Maps, Mapquest) and the terms of use for your map base layer must be compatible with our CC BY 4.0 license.

Note: if you created the map in a software program like R or ArcGIS, please locate and indicate the source of the basemap shapefile onto which data has been plotted.

If your map was obtained from a copyrighted source please amend the figure so that the base map used is from an openly available source. Alternatively, please provide explicit written permission from the copyright holder granting you the right to publish the material under our CC BY 4.0 license.

If you are unsure whether you can use a map or not, please do reach out and we will be able to help you. The following websites are good examples of where you can source open access or public domain maps:
* U.S. Geological Survey (USGS) - All maps are in the public domain. (<http://www.usgs.gov>)
* PlaniGlobe - All maps are published under a Creative Commons license so please cite u201cPlaniGlobe, <http://www.planiglobe.com>, CC BY 2.0u201d in the image credit after the caption. (<http://www.planiglobe.com/?lang=enl>)
* Natural Earth - All maps are public domain. (<http://www.naturalearthdata.com/about/terms-of-use/>).

We have updated the figure legends to include the direct links to the shapefiles and the license information: “The base layer of the map was sourced from GADM (<https://gadm.org/download_country.html> and <https://gadm.org/license.html>)”.

5) In the online submission form, you indicated that "will be made available on request following institutional policy governing data sharing". All PLOS journals now require all data underlying the findings described in their manuscript to be freely available to other researchers, either
- In a public repository
- Within the manuscript itself
- Uploaded as supplementary information.

This policy applies to all data except where public deposition would breach compliance with the protocol approved by your research ethics board. If your data cannot be made publicly available for ethical or legal reasons (e.g., public availability would compromise patient privacy), please explain your reasons by return email and your exemption request will be escalated to the editor for approval. Your exemption request will be handled independently and will not hold up the peer review process, but will need to be resolved should your manuscript be accepted for publication. One of the Editorial team will then be in touch if there are any issues.

We have updated the data accession statement as follows:

Hand, foot and mouth disease (HFMD) surveillance data is curated and stored by the National HFMD Surveillance System of Malaysia, under the Ministry of Health and available on the government website data.org.my at <https://archive.data.gov.my/data/ms_MY/dataset/bilangan-kes-penyakit-hfmd-mingguan-mengikut-negeri>. Code to perform the statistical analyses is freely available in the following GitHub repository <https://github.com/Tori-Cox/Malaysia_HFMD_Rt>.

6) Please ensure that the funders and grant numbers match between the Financial Disclosure field and the Funding Information tab in your submission form. Note that the funders must be provided in the same order in both places as well. State what role the funders took in the study. If the funders had no role in your study, please state: "The funders had no role in study design, data collection and analysis, decision to publish, or preparation of the manuscript.".
If you did not receive any funding for this study, please simply state: u201cThe authors received no specific funding for this work.u201d

It has been checked.

**Summary and General Comments**:

**Reviewer #1:** This is an interesting epidemiological study that analysed 11 years of surveillance data in Malaysia to investigate the transmission factors of HFMDs, and is well suited to the scope of PLOS Neglected Tropical Diseases. Firstly, I would like to congratulate the authors for piecing together and evaluating an extensive data set. Overall, I very much enjoyed reading this manuscript. The manuscript is well written, proper statistical methods of analysis have been carried out and well explained. I do have a few comments that I hope will help improving the manuscript, or maybe just give the authors food for thoughts.

We thank the reviewer for the positive evaluation and insightful comments.

L174-207: The authors' multivariate analysis is commendable for employing an indicator that precisely quantifies the spread of infectious diseases through the evaluation of Rt. Nevertheless, there remains a concern that the results might vary depending on the methodology used to input the explanatory variables. Ensuring the robustness of the primary findings through comprehensive sensitivity analyses would enhance their reliability. For instance, meteorological factors such as mean temperature, relative humidity, and precipitation could exhibit lag delayed effects**.** Additionally, it is important to question the assumption of linearity in the exposure-response relationship, thereby justifying the use of a linear mixed-effects regression model.

Thank you for these suggestions. We have explored lag delayed effects up to 14 days (please see the response to the point 3 below). In addition, we have now performed a sensitivity analysis testing the meteorological variables as non-linear effects in the univariable regression models. Each variable was categorised according to its 10th to 90th percentiles and added to the baseline model as a random effect categorical variable. The linearity of the regression coefficient pattern over the percentiles was assessed and linear relationships with HFMD Rt were deemed suitable for the meteorological variables in the main analysis. We have added a paragraph at the end of the Methods section describing this analysis, and a new Figure S24 showing the regression coefficients of this sensitivity analysis.

L174-207: The evaluation of the predictive capability of statistical models is essential. Empirical validation requires an assessment of the model's fit to the data and the control of time-series trends.

To validate the model, we have now computed the marginal R-squared and performed an analysis of the residuals. We have added the following text to the Results section:

*[…] As a result, the marginal R-squared, which is an estimate of how much variance in Rt is explained by the predictors of the model (i.e. the fixed effects), was low, of only 0.22. However, the model fitted well the magnitude and the trend of Rt, as supported by the analysis of the residuals. Indeed, the residuals were centred around zero (S18 Fig) and the detrended series looked stationary (S19-S22 Figs), with no pattern or trend over time. […]*

L363: While the authors have assumed a Gaussian distribution, it would be prudent to assess the fit against alternative distributions, such as quasi-Poisson or negative binomial, to ensure the most appropriate model is employed.

Our dependent variable is the effective reproduction number (Rt), which is a continuous variable (please see Figures S7-S11). We believe that it would not make sense to use a Poisson or Negative Binomial to model Rt, as these are discrete distributions.

L385: The examination of delayed effects of meteorological factors should consider a broader range of lags. Given the incubation period of HFMD, an evaluation extending up to 7 to 14 days would be advisable.

Thank you for this suggestion. We have now tested the proportion of school holiday days and the meteorological variables at lags up to 14 days. Please see the updated Supplementary Table S2 and Supplementary Figure S12 for the new univariable results, and Table 1, Supplementary Table S3, and Supplementary Figure S13 for the new multivariable model results.

The new final multivariable model has only slightly changed: now it includes cumulative rainfall at a lag of 14 days with a negative effect on Rt, instead of a lag of 4 days with a positive effect. However, this effect is again very small. Overall, our main conclusions remain the same.

L391: If R-INLA is used, it should be evaluated using multiple indices, not just WAIC. For example, it may be necessary to thoroughly examine models with increased complexity regarding input variables and model structure, such as the deviance information criterion and average cross-validation log score.

The models and manuscript have been updated to evaluate the variable selection steps using both the WAIC and the DIC indices. These are reported in Tables S2 and S3, and in S13 Fig. The ranking of univariable models obtained with the DIC is similar to that obtained with the WAIC (Table S2). In particular, the two indices provide the same final multivariable model (Table S3).

Dataset and code sharing: It may be a good idea to include the dataset used in this study and the code to reproduce the results in the supplementary materials, so that third parties can reproduce the study. Also, although we were unable to access the Github repository provided by the authors (and so were unable to evaluate its contents), is there a reason for this?

We have updated the data accession statement:

Hand, foot and mouth disease (HFMD) surveillance data is curated and stored by the National HFMD Surveillance System of Malaysia, under the Ministry of Health and available on the government website data.org.my at <https://archive.data.gov.my/data/ms_MY/dataset/bilangan-kes-penyakit-hfmd-mingguan-mengikut-negeri>.

The GitHub repository with the code used for the analyses is now publicly accessible.

Discussion: It is suggested that a section detailing the study's limitations be included, highlighting three to five key limitations and providing a concise summary of potential avenues for future research.

The Reviewer is right, the Discussion does not include a specific paragraph of limitations. However, limitations and avenues for future research are included in the Discussion for each of the main analyses and results of the manuscript. Briefly, these include: the need to further explore the existence of potential waves of HFMD spread at a local level (paragraph 4 of the Discussion); no data available on reactive school closure (paragraph 6 of the Discussion), which may result on an underestimation of the effect of this variable on transmission; and the fact that we did not model changes in serotype-specific population immunity (paragraph 7 of the Discussion).

We have also added a new paragraph to the Discussion stating ongoing work that extends the current analysis over the COVID-19 years. This reads:

*We purposely excluded the COVID-19 years in the current manuscript, to avoid confounding effects of the impact of non-pharmaceutical interventions on HFMD transmission. In 2022, Malaysia experienced one of the largest HFMD outbreaks recorded in the country. Future work should focus on quantifying the impact of control interventions against COVID-19 on HFMD dynamics.*

**Reviewer #2:** This paper delves into the epidemiological traits of HFMD in Malaysia and scrutinizes the influence of meteorological factors on its prevalence. As a comprehensive study, it holds considerable significance. Nevertheless, there are a few areas for improvement:

1. The dataset spans from 2009 to 2019. Incorporating data from 2020 to 2024 would enrich the analysis, particularly by illuminating the impact of the COVID-19 pandemic on HFMD trends.

We thank the reviewer for the suggestion. We are currently working on another piece of work that looks specifically at the impact of COVID-19 on HFMD transmission dynamics in Malaysia. We have added the following paragraph to the Discussion:

*We purposely excluded the COVID-19 years in the current manuscript, to avoid confounding effects of the impact of non-pharmaceutical interventions on HFMD transmission.* *In 2022, Malaysia experienced one of the largest HFMD outbreaks recorded in the country. Future work should focus on quantifying the impact of control interventions against COVID-19 on HFMD dynamics.*

Furthermore, in recent years, typing efforts have improved, and in our ongoing work on the impact of COVID-19 on HFMD dynamics we are able to employ a transmission model calibrated to time-series of HFMD cases and typing data, which is not possible for the years covered in the current manuscript.

2. Conducting a genetic evolutionary analysis of prevalent enteroviruses in Malaysia, such as EV-A71, could provide deeper insights into the virus's mutations and potential implications for HFMD.

We thank the reviewer for the suggestion. The main aim of this study was to describe the spatio-temporal dynamics of HFMD cases and assess factors associated with transmission. In Malaysia, HFMD is a notifiable disease, and therefore syndromic surveillance data is incredibly rich and quite exhaustive, as seen in this current analysis, based on >300.000 cases reported over the 11-year period. However, typing and sequencing of HFMD cases was very limited before 2017, and although it has improved recently, the numbers remain low. Therefore, genetic data would not be the most suitable to study HFMD epidemic dynamics in our period of interest.

On a related note, there is a paragraph in the Discussion that states the limitation of having limited serotype-specific data:

*Previous studies have shown the importance of the building up of serotype-specific population immunity and subsequent replenishment of susceptible individuals in driving the cyclical patterns of HFMD epidemics [2], [9]. However, information on circulating serotypes over the study period was very scarce, not available for all years, and not based on systematic testing. Therefore, we were unable to account for changes in serotype-specific population immunity in the analysis of drivers of transmission. To counter this and as done in previous work [10], we added a variable that measured the cumulative incidence of cases during each epidemic period and included this variable in the baseline model. However, although this approach may account for the depletion of susceptible during a given epidemic, it is unable to capture effects on a longer time scale of years.*
